# Estimating the treatment effect from non-randomized studies: The example of reduced intensity conditioning allogeneic stem cell transplantation in hematological diseases

**DOI:** 10.1186/1471-2326-12-10

**Published:** 2012-08-16

**Authors:** Matthieu Resche-Rigon, Romain Pirracchio, Marie Robin, Regis Peffault De Latour, David Sibon, Lionel Ades, Patricia Ribaud, Jean-Paul Fermand, Catherine Thieblemont, Gérard Socié, Sylvie Chevret

**Affiliations:** 1 Département de Biostatistique et Informatique Médicale, Hôpital Saint-Louis, AP-HP, Paris 75010, France; 2 INSERM, UMRS 717, Paris 75010, France; 3 Université Denis Diderot Paris 7, Paris 75010, France; 4 Service d’Hématologie Greffe, Hôpital Saint-Louis, AP-HP, Paris 75010, France; 5 Service d'Hématologie Clinique, Hôpital Avicenne, AP-HP, Bobigny 93100, France; 6 Service d'Immuno-Hématologie, Hôpital Saint-Louis, AP-HP, Paris 75010, France; 7 Service d'Onco-Hématologie, Hôpital Saint-Louis, AP-HP, Paris 75010, France

**Keywords:** Propensity score, Allogeneic stem cell transplantation, Treatment effect, Non-randomized studies

## Abstract

**Background:**

In some clinical situations, for which RCT are rare or impossible, the majority of the evidence comes from observational studies, but standard estimations could be biased because they ignore covariates that confound treatment decisions and outcomes.

**Methods:**

Three observational studies were conducted to assess the benefit of Allo-SCT in hematological malignancies of multiple myeloma, follicular lymphoma and Hodgkin’s disease. Two statistical analyses were performed: the propensity score (PS) matching approach and the inverse probability weighting (IPW) approach.

**Results:**

Based on PS-matched samples, a survival benefit in MM patients treated by Allo-SCT, as compared to similar non-allo treated patients, was observed with an HR of death at 0.35 (95%CI: 0.14-0.88). Similar results were observed in HD, 0.23 (0.07-0.80) but not in FL, 1.28 (0.43-3.77). Estimated benefits of Allo-SCT for the original population using IPW were erased in HR for death at 0.72 (0.37-1.39) for MM patients, 0.60 (0.19-1.89) for HD patients, and 2.02 (0.88-4.66) for FL patients.

**Conclusion:**

Differences in estimated benefits rely on whether the underlying population to which they apply is an ideal randomized experimental population (PS) or the original population (IPW). These useful methods should be employed when assessing the effects of innovative treatment in non-randomized experiments.

## Background

Randomized controlled trial (RCT) is considered the gold standard study design for removing sources of bias from observations when estimating the effects of a treatment [1,2]. However, in some situations, it may be difficult, unnecessary, inappropriate, or impossible to perform an RCT [3], and the majority of the evidence comes from observational studies [4,5].

This is notably true when evaluating non-myeloablative or reduced-intensity conditioning (RIC) regimens before allogeneic stem cell transplantation (Allo-SCT). RIC Allo-SCT has emerged in the last decade as an attractive modality to decrease transplant-related toxicity. The enthusiasm for this technique has been based on heterogeneous observational studies ranging from case reports to registry cohort studies [6-14]. These studies are very heterogeneous in terms of patient selection criteria and outcomes, RIC regimens and timing. For this reason, conclusions regarding the the overall body of evidence in this area are very limited [15]. Only a few prospective controlled clinical trials have been performed in studies of myeloma. This is mostly due to practical difficulties and selection restrictions for patients affected by advanced or refractory diseases, elderly patients, or patients with comorbidities for whom no other treatment option could be clearly proposed. In these few recent prospective non-randomized studies that have been conducted [16-18], the availability of an HLA-identical or non-identical sibling donor has been considered equivalent to so-called ”genetic randomization” of bone marrow transplant (BMT) against chemotherapy, justifying the absence of RCT [19-21]. Nevertheless, results of such studies are still vulnerable to selection bias and confounding factors.

In RCTs, the use of inclusion and exclusion criteria yields a sample of subjects that are all eligible for each of the treatments under study. By contrast, in observational studies, baseline selection criteria differing between Allo-SCT and other treatments may also affect patient outcome and lead to bias in the estimated effect of [2,22]. Thus, non-randomized comparative designs expose to unequal distributions of covariates that impact both the outcome and the decision to treat, so-called ”confounding by indication” [23]. Adjusted techniques of treatment estimation through the use of multivariate regression models have been widely used to control for confounding in observational data, but these methods do not provide any causal evidence comparable to that derived from RCTs. Formally, an association is considered causal when the observed outcome under the studied exposition is different from what would have been observed in the absence of the exposition. Because the latter outcome cannot actually happen, it is generally known as a counterfactual outcome [24]. In an ideal randomized design with blind assignment, full compliance, and no loss during follow-up, the absence of confounding data ensures that treated and non-treated patients exchangeable. In this setting, RCT allows causal claims about the population in the study to be deduced from differences between the treatment groups [25]. By contrast, in observational studies, because treated and non-treated populations are not exchangeable, no causal evidence could be derived from the original data [26]. Therefore, specific statistical tools have been developed to enable appopriate causal conclusions to be derived from observational data. These tools re-create the conditions of conditional exchangeability as observed in an RCT.

**Figure 1:**
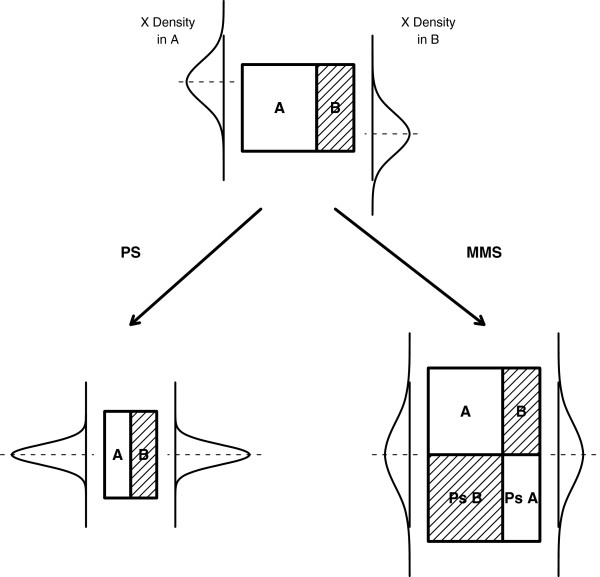
**Illustration of the different distributions of a covariate (X) in two non-randomized samples (A & B). **The propensity score method (PS) aims at re-creating the conditions of a pseudorandomization, while the inverse probability weighting (IPW) approach aims at re-creating a pseudopopulation where patients A and B are exchangeable. Both methods aim at obtaining a similar distribution of the covariate *X* in the two groups.

This article provides an illustration of two of these specific statistical approaches in the particular setting of Allo-SCT evaluation of observational cohorts. The methods described here aim at handling confounding variables induced by non-randomized designs, namely, the propensity score-based (PS) matching approach [27] and the inverse probability of treatment weighting (IPW) approach, which is derived from the marginal structural models [28]. These statistical methods have both been developed to re-create exchangeability in the presence of all confounding variables. By re-creating populations in which all the confounding variables have comparable distributions (Figure 1), they allow a causal inference and unbiased estimation of treatment effect [26,29].

## Methods

### The Allogeneic Stem Cell Transplantation cohorts

Allogeneic Stem Cell Transplantation (Allo-SCT) was performed in patients who relapsed after autologous transplantation (in Saint-Louis Hospital, Paris, France) but remained chemosensitive. Among them, all consecutive patients with multiple myeloma (MM, 23 pts), follicular lymphoma (FL, 28 pts) or Hodgkin’s disease (HD, 31 pts), were considered for analysis as follows.

• MM: Between October 2002 and August 2006, 23 consecutive MM patients under 60 years of age and in their first or second relapse received RIC Allo-SCT.

• FL: All 28 consecutive patients who received Allo-SCT for relapsing/refractory FL from December 1989 to January 2007 were eligible for analysis.

• HD: A total of 31 HD patients who received Allo-SCT from January 1995 to December 2008 were consecutively analyzed.

### Selection of controls

The main issue in observational studies is the definition of control subjects to whom comparison of outcomes can be applied. As reported by Austin [30], observational studies should be designed to approximate randomized experiments as closely as possible. This suggests that particular attention should be paid to include only those subjects who are eligible to receive either treatment or intervention [31]. This refers to the “positivity” or "overlap" [32] assumption and requires a careful selection of the original cohorts of untreated patients.

**Figure 2:**
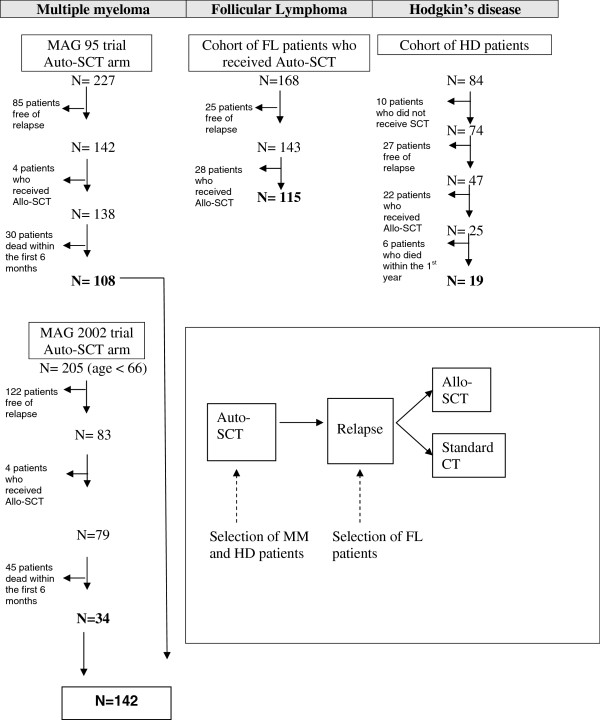
Flow chart for the selection of the control patients.

As summarized in the flow chart depicted in Figure 2, controls were selected carefully. MM controls were selected from patients enrolled in the MAG-95 and MAG-2002 trials [33], while FL and HD patients were selected from hospital cohorts. The clinical trials from which the Multiple Myeloma control patients were selected, have been carried out in compliance with the Helsinki Declaration and French laws regarding biomedical research at the time the trials were conducted. In particular the studies were approved by the Ethics Committee of Saint Louis Hospital (Paris, France). To insure the validity of the overlap assumption, we restricted the controls to patients who survived at least six months after relapse (MM) or one year after auto-SCT (HD), since this was the minimal time between relapse or first Auto-SCT and Allo-SCT in MM and HD patients from the Allo-SCT groups, respectively.

Three cohorts comprised of 276 patients (142 MM, 115 FL and 19 HD) who relapsed after autologous transplantation (auto-SCT) but did not undergo allogeneic stem cell transplantation were retained for analysis. Patients who had contraindications (severe comorbidities, age > 65 years..) to Allo-SCT were excluded from the cohort.

To estimate the benefit of Allo-SCT from observational cohort data, three analyses were performed in each cohort of MM, FL and HD patients separately. Both approaches require modeling the probability of being treated.

### Probability of treatment model: Propensity Score

The propensity score (*PS*) is derived from the probability that a given patient would receive Allo-SCT conditionally to his confounding covariates, *X*. It is estimated by fitting a multivariate logistic model to the original cohorts of treated and untreated patients in order to predict allocation to Allo-SCT from patient covariates, *X*[27,34,35]. This aims to re-create exchangeability, that is, there is no unmeasured confounding variable. Unfortunately, this assumption cannot be tested, and the PS model requires the analyst to have confidence that *X* contains almost all characteristics related to both treatment and outcome, and that there are no additional, unmeasured, confounders [36].

Since one cannot know all the covariates that are confounding, this multivariable model should include most of the covariates measured at baseline, or at least those known or suspected to be confounding, in the hope that there is at least one measured covariate strongly related to all the confounders [37,38]. Nevertheless, due to the sample size of the cohorts, we only included those variables that were strongly related to the treatment allocation in the PS models [38]. These included age at diagnosis, time to relapse and beta-2-microglobulin level for the MM cohorts, age at relapse, time from relapse to SCT and number of previous regimens for the FL cohorts, and age at diagnosis and stage for the HD cohort.

### Estimation of causal benefit of Allo-SCT

The main endpoints were overall survival (OS) and event-free survival (EFS). These were defined in the Allo-SCT groups from the date of Allo-SCT for MM and FL and from the date of first autologous SCT for HD. In the non-Allo-SCT patients, OS and EFS were defined from the date of relapse plus six months for MM, from the date to autologous SCT for FL and from the date of first autologous SCT plus 12 months for HD. We first fitted standard Cox models to the original samples. Then, specific methods to handle confounding variables were applied.

#### Matched propensity score-based approach

Propensity score (PS) analysis attempts to create a comparison group of non-treated patients that closely mimics the group of treated patients by matching based on the likelihood that a given patient has received Allo-SCT considering all his confounders (Figure 1) [34].

It is based on a matched-paired analysis as follows [39,40]: Allo-SCT patients and controls are matched on the logit of the *PS* using calipers of width equal to 0.2 of its standard deviation (SD). Two patients of a pair cannot differ in the linear score of being treated by more than 0.2 SD [39,40]. A nearest-neighbor matching algorithm was thus used to form pairs of treated and untreated subjects with the constraint that once a patient had been matched, he(she) could not be further matched.

The degree to which the matching procedure adequately balanced covariates between patients who received Allo-SCT and those who did not was evaluated by comparing the standardized mean differences of the main measured baseline covariates between treated and untreated patients in the original and matched samples [35,41].

The benefit of Allo-SCT to outcome was then estimated by fitting a Cox model that applies to the propensity-based matched sample using a robust variance estimator to take into account the correlation induced by the matching [42,43].

#### Inverse probability weighting approach

As an alternative to the PS matching approach, inverse probability of treatment weighted (IPW) estimators have been developed to draw causal conclusions from observational data in the presence of confounding variables by indication [24,44,45]. This approach consists of creating a hypothetical population, the so-called *pseudo population*, that includes patients for which there are no example of Allo-SCT treated or untreated patients sharing the same characteristics (Figure 1) [28,46,47]. In that *pseudo population*, in which the probability of treatment no longer depends on covariates, the effect of the treatment on outcome is the same as in the original selected population. This *pseudo-population* is expected to have the *X* distribution of the total population.

This method uses propensity scores to derive weights for individual observations. Actually, each individual is assigned a weight, which is inversely proportional to his (her) probability of receiving the treatment he (she) actually received (either Allo-SCT or not), conditionally to the value of his (her) counfonding covariate *X*[28]. It is thus computed directly from 1/*PS* or 1/(1-*PS*), respectively. This is also referred as the "PS weighted modelling method" or the "inverse propensity weighted method" [28,29,36,46,48].

A marginal causal effect of Allo-SCT on survival or EFS in the resulting *pseudo-cohorts* is then analyzed by using a weighted Cox proportional hazard model. As in the matched propensity score-based approach, a robust variance estimator is applied to take into account that each patient contributed more than once, given that weights are not equal to one [28].

### Statistical analysis

Logistic models, Cox models and weighted Cox models were fitted using standard packages of R software [49]. Matching was performed using the *Matching* R package. Equivalent packages are available in standard statistical softwares.

We checked for model misspecifications, *i.e.*, of either the PS or IPW models. For the PS model, we checked for linearity between continuous covariates and the log-odds of receiving treatment [41]. For the IPW model, we explored the distribution of weights (mean, standard deviations, minimum and maximum) [39]. Weights distribution was considered as optimized when mean weights were close to 1 with limited dispersion [28,46]. Reductions in the imbalances reached by each method were assessed using graphical displays of the standardized mean difference in main covariates between treatment groups [41,50].

Finally, Cox model assumptions of proportional hazards and log-linearity for continuous covariates were checked [51].

## Results and discussion

Three separate analyses were thus performed corresponding to MM, FL and HD patients, respectively.

### Baseline comparison

**Figure 3:**
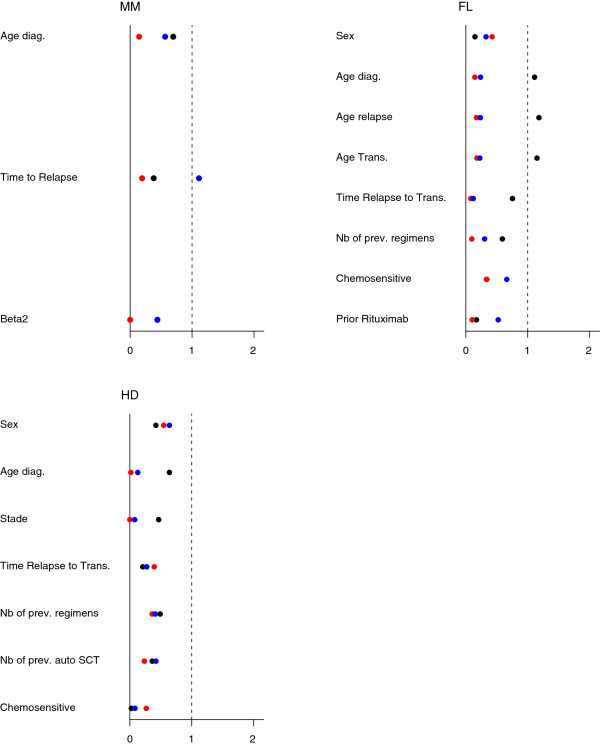
**Imbalances in the MM, FL and HD cohorts, defined as the standardized means differences of covariate values between the two treatment groups. **· Naive Analysis, red circle symbol = Propensity Score Model, blue circle symbol = Marginal Structural Model.

As expected due to the-non randomized designs, and although controls were selected carefully to avoid non-overlapped confounding variables, Allo-SCT and control patients markedly differed at baseline (Table 1). As expected, all patients who received Allo-SCT were younger than those who did not. Moreover, MM patients who received Allo-SCT had relapsed earlier (median 16 vs. 26.5) than those who did not; by contrast, HD patients from the Allo-SCT group had delayed relapse as compared to the control group (median: 1.2 vs. 2.4). Otherwise, FL patients from the Allo-SCT group received a higher number of previous regimens (4 vs. 3) while those HD patients had less (3 vs. 4). This is illustrated on plots of absolute mean standardized differences in Figure 3.

**Table 1:** Main characteristics of patients according to treatment group before and after matching or weighting

**Median [Q1-Q3]**	**Allo-SCT**	**Controls**	**p-value**
**N (%)**			
**MM**			
**Original set**	**n=23**	**n=142**	
Age	48 [40.5-51]	51.5 [47-55]	0.005
Beta2 ≥ 3.5	4 (17 %)	52 (37 %)	0.12
Months to relapse	16 [11–32.5]	26.5 [17-38]	0.014
Matched set	n=21	n=21	
Age	49 [41-51]	46 [42-50]	0.24
Beta2 ≥ 3.5	4 (19 %)	4 (19 %)	0.71
Months to relapse	17 [13-33]	24 [17-32]	0.22
**Weighted set**	**n=268**	**n=165**	
Age	56 [51-58]	51 [46-54]	0.15
Beta2 ≥ 3.5	4 (19%)	4 (19%)	0.28
Months to relapse	58 [26–70]	25 [17-36]	0.08
**FL**			
**Original set**	**n=28**	**n=115**	
Age	38 [33-42]	46 [40-52]	0.0001
No previous regimens	4 [3,4]	3 [2-4]	0.005
Months to relapse	6.7 [5.6-9.2]	4.6 [3.7-6.1]	0.0001
**Matched set**	**n=19**	**n=19**	
Age	38 [33-42]	38 [33-45]	0.35
No previous regimens	3 [3,4]	3 [3,4]	0.90
Months to relapse	6.3 [4.7-8.9]	7.8 [4.0-10.7]	0.82
**Weighted set**	**n=78**	**n=117**	
Age	39 [35-46]	44 [34-50]	0.42
No previous regimens	3 [3,4]	3 [2-4]	0.16
Months to relapse	5.9 [4.7-7.4]	5.3 [3.8-9.3]	0.03
**Hodgkin disease**			
**Original set**	**n=23**	**n=19**	
Age	23 [19-29]	29 [24-35]	0.05
No previous regimens	4 [3,4]	4 [4,5]	0.05
Months to relapse	1.2 [0–9.2]	2.4 [0–7.2]	0.94
**Matched set**	**n=15**	**n=15**	
Age	24 [21-31]	25 [23-30]	0.98
No previous regimens	3 [3,4]	4 [4,5]	0.21
Months to relapse	1.9 [0.3-9.9]	1.8 [0–5.4]	0.49
**Weighted set**	**n=41**	**n=40**	
Age	26 [20-49]	25 [20-32]	0.71
No previous regimens	3 [3,4]	4 [4]	0.17
Months to relapse	0.1 [0–0.7]	0.1 [0–0.5]	0.35

**Table 2:** Estimated hazard ratio (HR) of death or event and 95% confidence interval using naive, matched propensity score-based or IPW approaches

	**Numbers of patients**		**OS : HR (CI95%)**			**EFS : HR (CI95%)**		
	**Allo-SCT/Controls**							
	**Original samples**	**PS-matched samples**	**Naive**	**PS**	**IPW**	**Naive**	**PS**	**IPW**
**MM**	23/142	21/21	0.38 (0.18;0.80)	0.35 (0.14;0.88)	0.72 (0.37;1.39)			
**FL**	28/115	19/19	2.55 (1.37;4.75)	1.28 (0.43;3.77)	2.02 (0.88;4.66)	1.21 (0.68;2.18)	0.45 (0.17;1.21)	0.67 (0.31;1.41)
**HD**	22/19	15/15	0.33 (0.12;0.87)	0.23 (0.07;0.80)	0.60 (0.19;1.89)	0.71 (0.38;1.35)	0.47 (0.20;1.09)	0.64 (0.33;1.22)

### Treatment effect

From the naive analyses based on standard Cox models, a significant benefit associated with RIC Allo-SCT was observed for MM patients with an estimated hazard ratio (HR) of death at 0.38 (95% confidence interval 95%CI: 0.18;0.80) and for HD patients (HR = 0.33, 95%CI: 0.12;0.87) while Allo-SCT seemed to be deleterious in FL patients (HR = 2.55, 95%CI: 1.37;4.75). No significant benefit was found in terms of EFS (HR =1.21, 95%CI: 0.68;2.18, HR =0.71, 95%CI: 0.38;1.35 for FL and HD respectively).

#### Matched propensity score-based approach

The matching procedure resulted in a drastic reduction of the sample size of the PS-matched samples. From the original datasets, 21 (91% of RIC Allo-SCT patients, 15% of controls) matched pairs could be constituted from MM patients, as compared to 19 (68% of Allo-SCTpatients, 17% of controls) from the the FL patients, and 15 (48% of the Allo-SCT patients and 79% of the controls) from the HD patients. This relies both on the original differences in sample sizes and the non-overlapped covariates values (Table 1). As a result, baseline imbalances between the two matched sets were reduced (Figure 3). Note that imbalance was also reduced for those covariates not included in the PS, especially age at diagnosis and age at transplantation in the FL cohort.

Based on these PS-matched samples, we observed a significant benefit to the survival of Allo-SCT as compared to non Allo-SCT MM patients with an estimated HR of death at 0.35 (95%CI: 0.14-0.88), as well as HD (HR = 0.23, 95%CI: 0.07;0.80). A similar result was not found for FL patients (HR = 1.28; 95%CI: 0.43;3.77). No significant benefit was found for EFS with the estimated HR of event at 0.45 (95%CI: 0.17;1.21) in FL and 0.47 (95%CI: 0.20;1.09) in HD.

#### IPW approach

Using the IPW approach, imbalances in the pseudo-cohorts were also reduced, though reduction was slightly less effective than that observed using the PS (Figure 3). Actually, the distribution of the covariates in the weighted samples (*pseudo-population*, was close to that observed in the original datasets (Table 1).

Despite similar trends, the survival benefit associated with Allo-SCT in MM and HD patients was erased using IPW based analyses as compared to PS-based analyses, which yielded an estimated HR of death of 0.72 (95%CI: 0.37-1.39) and 0.60 (95%CI: 0.19-1.89), respectively. Results for FL patients remained non-significant (HR = 2.02, 95%CI: 0.88;4.66). No significant benefit was found for EFS, which gave an estimated HR of event of 0.67 (95%CI: 0.31;1.41) in FL and 0.64 (95%CI: 0.33;1.22) in HD.

The main objective of this paper was to report examples of treatment estimation from observational cohorts in the particular setting of Allogeneic Stem Cell Transplantation. Despite the fact that the randomized controlled trial (RCT) is the gold standard for removal of most sources of bias from observational data, such studies are difficult to conduct when evaluating Allo-SCT. In situations such as HLA-matched sibling allogeneic transplants, some authors have advocated a biological assignment trial [16]. Such trials are also known as *genetic* or *Mendelian randomization* trials, and these trials consider the selection of the sibling donor and recipient genes from their parents as a random process at the time of conception. Nevertheless, implementing such a trial requires careful consideration of the ethical issues and potential biases (prognostic factor imbalance, enrollment bias) [21]. Moreover, these trials are prospective and require several years to provide estimates of survival benefits, while observational information about treatment effect are already available.

Indeed, observational studies have several advantages over randomized, controlled trials, including lower cost, greater timeliness, and a broader range of patients [8]. Moreover, systematic reviews tend to demonstrate that, when adequaltely performed, observational studies give results similar to those of randomized clinical trials [52]. In the hematology field, and especially in that of Allo-SCT, many international cooperating groups exist and register all blood or marrow transplantation experiments. Notably, the European Group for Blood and Marrow Transplantation (EBMT) and the Center for International Blood and Marrow Transplant Research (CIBMTR) have collected information about patients undergoing Allo-SCT since the 1970s. Such observational registers could be a an important source of information when estimating the causal effect of Allo-SCT as compared to autologous SCT or other standard treatments. Nevertheless, standard statistical analyses from such observational data may result in biased and associational rather than causal estimates of treatment effect [27,28].

Since 2000, there has been a growing interest in the use of statistical methods to estimate unbiased treatment effects from observational studies and begin to be used in haematology or oncology [53-56]. Most of these methods are based on the propensity score, *i.e.* re-creation of the exchangeability between the two treatments groups. Two main approaches have been proposed in this setting, namely, the propensity score-matched approach and the inverse probability weighting approach [36]. If these approaches were initially proposed for large studies, recent work by Pirracchio et al. showed that propensity score approaches (matching or IPW) are also valid and useful on small sample studies [5]. We illustrated how those methods could perform to estimate the effect of Allo-SCT on survival and event-free survival using observational data from multiple myeloma, follicular lymphoma and Hodgkin’s disease observational cohorts. Obviously, considering our low sample sizes, our findinds should be confirmed by larger studies.

However, as recently pointed out [32], both approaches are interested in estimating different quantities, namely the average treatment effect (ATE) and the ATE for the treated (ATT). The propensity based approach aims at estimating the ATT, *i.e.* the effect of treatment on those subjects who are treated, allowing observational studies to be designed similarly to randomized experiments [57]. By contrast, the inverse probability weighting approach aims at estimating the ATE, that is, the average effect on the population of moving all subjects from being untreated to treated. According to specific clinical contexts, researchers should determine the most clinically meaningful treatment effect. When evaluating the benefit of Allo-SCT as compared to chemotherapy, ATE (and thus, the IPW approach) would answer the question about how outcomes would change if a policy was instituted that all patients eligible for either therapy were offered Allo-SCT. By contrast, ATT would answer the question of what was the effect of treatment for those who selected a particular modality such as Allo-SCT. This explains why estimated resulting hazard ratio estimates differed between the two approaches. Indeed, by contrast to the PS-based approach, the IPW approach never showed a significant impact of Allo-SCT on overall survival or event-free survival. In other words, the benefit of Allo-SCT appeared to be restricted to treated patients, while no average benefit appeared to be expected in the whole eligible population for Allo-SCT. This is likely to rely on the fact that the benefit of Allo-SCT may be restricted to some subsets of patients that have been excluded by matching in the PS-matched analyses but maintened, and possibly heavily weighted, in the IPW method. This further highlights the importance of the positivity (overlap) assumption.

Indeed, whatever the approach, each subject is assumed to have a non-zero probability of receiving either treatment. This suggests that observational studies should be designed similar to RCTs. That is, subjects who are ineligible for at least one of the treatments should be excluded [32]. Actually, this was exemplified in our cohorts by the percentage of control patients who could not be matched, ranging from 21% in HD up to 85% in MM. Such percentages could be related to the differences in the criteria used to define controls. Moreover, it is assumed that all variables related to both outcomes and treatment assignments were introduced in the propensity score model [35]. Rubin suggested including only variables that are strongly related to the treatment allocation, while others have proposed the application of selection algorithms [37,58]. Our PS models were based on unbalanced characteristics with known clinical significance and the number of variables was limited by the sample size. Therefore, one cannot exclude that other confusing characteristics should have been included in the PS model.

Other methods could be proposed to estimate treatment effect in non-randomized studies. The most popular method consists in estimating treatment effects using adjustment on covariates with a multivariable regression model [5].The main limitation of this approach is that the treatment effect estimated is neither the ATE nor the ATT. Indeed, the treatment effect measured is conditional on the other covariates and then biased if used as an estimate of the ATE or ATT. Another emerging approach is the instrumental variable (IV) approach which is an econometric method used to remove the effects of hidden bias in observational studies [5]. An instrumental variable has 2 key characteristics: it is highly correlated with treatment and does not independently affect the outcome, so that it is not associated with measured or unmeasured patient health status. In our case, none available variable could be considered as an IV. Moreover, this approach hasn’t been validated on small samples. This should deserve further evaluation to be used in such clinical settings**.**

## Conclusion

In summary, it is expected that hematologists involved in clinical research will face an increasing need for methods such as those discussed here when assessing effects of innovative treatments based on cohorts or registries. Actually, though they do not replace randomized trials, these approaches have already been widely used in other medical settings such as cardiology or critical care [7,59]. This could be similar to what happened a decade ago with competing risks approaches in estimating the incidence of relapse. Whatever the statistical innovation, full understanding of the method is required. Notably, differences in the proposed methods should be anticipated by considering the population of interest for which the benefit is likely to apply. In other words, physicians and researchers should carefully assess whether they are interested in estimating the average treatment effect in the eligible population or only in those who were treated.

## Abbreviations

RCT: Randomized Controlled Trial; RIC: Reduced-Intensity Conditioning; SCT: Stem Cell Transplantation; Allo-SCT: Allogeneic Stem Cell Transplantation; BMT: Bone Marrow Transplant; PS: Propensity Score; IPW: Inverse Probability of treatment Weighting; MM: Multiple Myeloma; FL: Follicular Lymphoma; HD: Hodgkin’s Disease; ATE: Average Treatment Effect; ATT: Average Treatment effect for the Treated; OS: Overall Survival; EFS: Event-Free Survival; SD: Standard Deviation; HR: Hazard ratio; EBMT: European Group for Blood and Marrow Transplantation; CIBMTR: Center for International Blood and Marrow Transplant Research.

## Competing interests

The authors declare that they have no competing interests.

## Authors’ contributions

MRR, RP, SC participated in the design of the study, performed the statistical analysis and drafted the manuscript. MR, DS, JPF, CT, GS participated in the design of the study. All authors read and approved the final manuscript.

## Pre-publication history

The pre-publication history for this paper can be accessed here:

http://www.biomedcentral.com/1471-2326/12/10/prepub
